# Refractory Methemoglobinemia Secondary to Topical Dapsone With Subsequent Autoimmune Hemolytic Anemia

**DOI:** 10.7759/cureus.28811

**Published:** 2022-09-05

**Authors:** Catharine Cantrell, Vincent Costers, Chandler C Wilson, Christopher J Dudek, Justin K Arnold

**Affiliations:** 1 Emergency Medicine, Tampa General Hospital, Tampa, USA; 2 Pharmacy, Tampa General Hospital, Tampa, USA; 3 Emergency Medicine, University of South Florida Morsani College of Medicine, Tampa, USA

**Keywords:** ascorbic acid, pediatrics, methylene blue, methemoglobinemia, refractory methemoglobinemia, topical dapsone

## Abstract

We present a case of refractory methemoglobinemia with subsequent autoimmune hemolytic anemia in a young female after two days of topical dapsone use. A 15-year-old female with no known genetic risk factors was found to have a methemoglobinemia concentration of 37.1% after presenting with cyanosis, dyspnea, tachycardia, and oxygen saturation of 88% on 15 L of oxygen via a non-rebreather mask. Despite treatment with methylene blue, her methemoglobin concentrations continued to spike, requiring additional doses of methylene blue in addition to ascorbic acid and cimetidine. After discharge on the fourth day, she presented to another hospital with similar symptoms and was again found to have methemoglobinemia before developing autoimmune hemolytic anemia. This patient had no known underlying risk factors, including a normal BMI, normal renal function, two negative glucose-6-phosphate-dehydrogenase (G6PD) deficiency tests, and surprisingly a negative Coombs test. Although rare, particularly in the setting of topical dapsone use, methemoglobinemia remains an important consideration in the differential diagnosis of cyanosis and hypoxia, with early recognition by the emergency medicine physician being imperative for good patient outcomes.

## Introduction

Dapsone (diaminodiphenyl sulfone) is an antimicrobial agent from the sulfone family commonly used to treat diseases like leprosy, malaria and pneumocystis pneumonia, or toxoplasmosis in immunosuppressed patients [1]. Topically, dapsone is also used to treat a range of dermatological disorders, including acne vulgaris [2]. Dapsone has been frequently reported as a cause of methemoglobinemia, with an estimated incidence of up to 20% after oral administration [3]. Case reports indicate that methemoglobinemia may also occur after topical use [3,4]. Methemoglobinemia is functional anemia whose manifestation correlates to the blood concentration of methemoglobin. Concentrations over 15% are often associated with cyanosis, while levels between 25-45% are associated with headache, lethargy, tachycardia, weakness, and dizziness. At levels over 45%, symptoms include dyspnea, metabolic acidosis, cardiac dysrhythmias, heart failure, seizures, and coma, while levels exceeding 70% are associated with high levels of mortality [3]. Infrequent case reports have shown that patients with methemoglobinemia may go on to develop autoimmune hemolytic anemia following methemoglobinemia in the setting of patients both with and without glucose-6-phosphate-dehydrogenase (G6PD) deficiency [5,6]. The primary treatment of methemoglobinemia is methylene blue, with ascorbic acid serving as an additional reducing agent [7]. Cimetidine, a cytochrome P450 inhibitor that has been shown to reduce levels of methemoglobin in patients on oral dapsone, may additionally be used [8].

## Case presentation

A 15-year-old, visibly cyanotic female presented to the emergency department via emergency medical services with a seven-hour history of nausea, lightheadedness, palpitations, and gradually worsening dyspnea. At the time of presentation, her vital signs included a heart rate of 160 beats/minute, blood pressure of 125/78 mmHg, respiratory rate of 24 breaths/minute, and saturating 88% on 15 L oxygen per minute via a non-rebreather mask. Her temperature was 37.4° C. Her medications included daily sertraline as well as topical dapsone for acne. She had previously taken topical dapsone for acne, stopped, and resumed its use two days before the presentation. The patient had spent the day at a theme park and admitted to being dehydrated. She denied any fever or viral respiratory symptoms, history of deep vein thrombosis or pulmonary embolism, G6PD deficiency or hemolysis, oral contraceptive use, pregnancy, drugs, or alcohol. She had received two vaccinations (Pfizer) against COVID-19. On physical exam, her lungs were clear to auscultation bilaterally. The patient reported presenting to the emergency department with similar but less severe symptoms five months earlier. At that time, her workup was unremarkable, including a negative CT pulmonary angiogram, and she was discharged home after a one-night admission and started on sertraline for presumed anxiety. Blood gas with co-oximetry was not ordered during this admission. At this presentation, an arterial blood gas with co-oximetry revealed a methemoglobinemia concentration of 37.1%. 

After discussion with the pharmacist, the patient was given two grams of intravenous ascorbic acid due to the risk of serotonin syndrome, given her concurrent sertraline use. However, after a discussion of the risks and benefits with a toxicologist at the local poison control center, it was decided to start methylene blue at an initial dose of 1 mg/kg, discontinue sertraline, and observe for signs of serotonin syndrome. The risk would be highest during the first administration of methylene blue, with a subsequent decrease over time as the patient cleared sertraline. A repeat blood gas co-oximetry two hours after presentation showed a methemoglobinemia level of 12.8% with no signs of serotonin syndrome and improvement in the patient's dyspnea. The patient was admitted to the pediatric intensive care unit.

**Figure 1:**
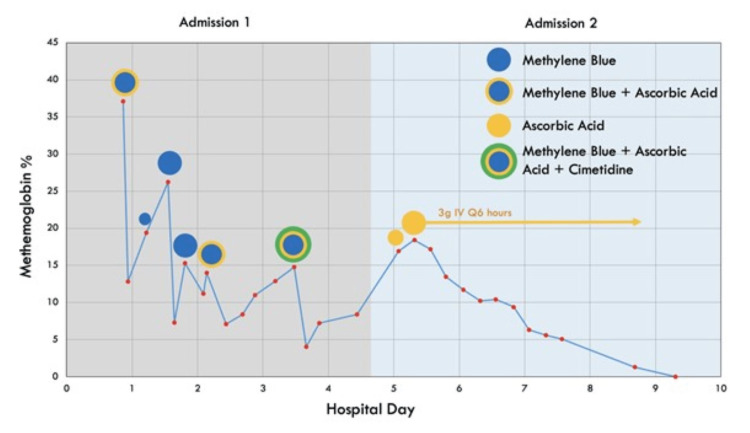
Hospital course

Three hours after the first dose of methylene blue was administered, the patient exhibited worsening respiratory distress, and her methemoglobin concentration was found to have increased to 19.4%. Despite a repeat dose of 0.5 mg/kg methylene blue, five hours later, her methemoglobin had risen to 26.2%, and she was again cyanotic, tachycardic, tachypneic, and saturating in the low 80% on a non-rebreather at 15 L oxygen per minute. At this time, her skin was decontaminated with soap and water to remove any residual dapsone cream, and an additional 1 mg/kg of methylene blue was given. Despite an initial decrease in her methemoglobin levels, within four hours, it was again measured at 15.3%. The patient denied applying dapsone during her hospital admission, and none was found in her belongings. Another 1 mg/kg dose of methylene blue was administered with a reduction in methemoglobin followed by another increase to 14% eight hours later. At that time, the patient was given both 1 mg/kg methylene blue with two grams of intravenous ascorbic acid to serve as an additional reducing agent. Thirty-two hours later, she was again found to have a methemoglobin concentration of 14%. A sixth and final dose of methylene blue was given at 1 mg/kg. In addition, 500 mg oral ascorbic acid and 400 mg cimetidine every six hours were ordered for the next seven days (Figure 1).

By the fourth day of admission, the patient's methemoglobin levels appeared to have stabilized under 10%, and she was asymptomatic. A G6PD test obtained during her hospital stay was negative for any deficiency. Following a discussion with the patient and her family, she was discharged home with one week of ascorbic acid and cimetidine therapy. Sertraline was resumed, and she was instructed to discontinue dapsone indefinitely.

However, later that same day, the patient presented to the emergency department pale and slightly cyanotic and complaining of dyspnea and "not feeling well". She was again found to have an elevated methemoglobin level of 16.9%, which had increased to 18.4% six hours later despite an initial dose of two grams of intravenous ascorbic acid. She was started on three grams of intravenous ascorbic acid every six hours. A second G6PD test during this stay was also confirmed to be negative for any deficiency. Over the next five days, the patient's methemoglobin level slowly trended down to 0.6% (Figure 1).

The patient then developed normocytic anemia, with her hemoglobin trending down from 11.9 g/dL at the time of admission to 7.4 g/dL six days later. She was given one unit of packed red blood cells on the 10th day of total admission with improvement in her hemoglobin but with worsening symptoms of anemia. The suspected hemolytic anemia was supported when further laboratory testing revealed elevated total bilirubin (peak 2.9 mg/dL), direct bilirubin (peak 0.34 mg/dL), lactate dehydrogenase (peak 509 units/L), reticulocytes (peak 10.4%), and a haptoglobin of <8 mg/dl (normal range 30-200 mg/dL). The total bilirubin at her first emergency department visit had been unremarkable at 0.9 on the day of admission. The patient was also found to have positive antinuclear antibodies (ANA, 1:640) with other elevated markers of autoimmune disease (Table 1).

**Table 1:** Autoimmune workup IgA - immunoglobulin A; IgG - immunoglobin G

Laboratory test	Units	Reference range	Result
Gliadin deaminated peptide IgA antibody	U/mL	0.0 - 6.9	14.0
Gliadin deaminated peptide IgG antibody	U/mL	0.0 - 6.9	51.0
Endomysial IgA antibody	–	Negative	Positive
Transglutaminase IgA antibody	U/mL	0.0 - 6.9	39
Transglutaminase IgG antibody	U/mL	0.0 - 6.9	7.2

Although the patient's Coombs test was negative after she was started on prednisone 40 mg twice a day, a marked increase was seen in her hemoglobin with a significant decrease in her bilirubin and lactate dehydrogenase. This supported the diagnosis of a true autoimmune hemolytic anemia despite the negative Coombs test. On the 17th day after the acute onset of her symptoms, the patient's hemoglobin stabilized, and her symptoms resolved. She was discharged home on prednisone 40 mg twice daily for 30 days with scheduled outpatient follow-up with hematology and rheumatology.

## Discussion

This case illustrates an interesting presentation of 10 days of persistent methemoglobinemia resulting from topical dapsone use with the subsequent development of autoimmune hemolytic anemia. While the incidence of methemoglobinemia from oral dapsone use is reported as high as 20%, methemoglobinemia from topical dapsone is relatively uncommon due to relatively poor absorption through the skin and appears only in case reports [3,4,9]. Additionally, prolonged methemoglobinemia from dapsone use has been reported in patients who were taking oral dapsone, which has a much higher level of systemic absorption in adipose tissue [10-12]. In a case reported by Wieringa et al., dapsone-associated methemoglobinemia was reported to have lasted for up to a year, although the patient was taking oral dapsone and was morbidly obese. In that case, the duration of methemoglobinemia was thought to be secondary to the drug depot in the adipose tissue [11]. The 15-year-old patient discussed in this case had a BMI of 22.8 kg/m^2^, normal renal function, and was using prescribed doses of topical dapsone.

The persistence of methemoglobinemia, in this case, may be explained by the long 48-hour half-life of topical dapsone with toxicity thought possible for up to five half-lives of the drug [9]. Another possible explanation for the refractory methemoglobinemia may be that the oxidative action of the methylene blue was causing reflexive methemoglobinemia. This has been previously described in patients who have G6PD deficiency but not in patients who have tested negative for the disease, as this patient did during her hospitalization [5,10]. Additionally, if the patient were experiencing significant oxidative stress from methylene blue, the lactate dehydrogenase would be expected to increase with subsequent doses. While no lactate dehydrogenase was drawn during the patient's initial admission, it was notably elevated two days after admission to the second hospital, although this lab was drawn for concern for hemolytic anemia, which would also cause the levels to be elevated. However, it should be noted that during the patient's second admission, her methemoglobinemia was treated only with ascorbic acid, and her methemoglobin levels trended down to almost zero without any further elevations.

In our case, the patient is also believed to have developed a subsequent autoimmune hemolytic anemia, which has been described only infrequently in the literature. In a case reported by Malkarnekar et al., two females were reported to have developed methemoglobinemia at different periods following ingestion of bio-nutrient plant compounds with suicidal intention. Both were found to be negative for G6PD deficiency. In each case, the patients developed subsequent hemolytic anemia approximately 72 hours after administration of methylene blue [5].

Additionally, while our patient tested negative for G6PD deficiency, autoimmune hemolytic anemia has been seen up to five months after a bout of methemoglobinemia in a patient that tested negative for G6PD deficiency [6]. This may be due to reticulocytosis during an acute hemolytic episode falsely elevating the patient's G6PD levels over baseline values. It is, therefore, important to be aware of possible false-negative tests for G6PD deficiency during a hemolytic crisis and to retest again in the future. In this case, however, the patient's total bilirubin level at the time of the first negative G6PD deficiency test was unremarkable, suggesting that no hemolytic crisis was present at that time.

Lastly, this case highlights a likely failure to recognize a case of methemoglobin during the patient's initial visit for similar symptoms five months earlier. At that time, the patient complained of chest tightness, dyspnea, and lightheadedness. While a blood gas was ordered, a co-oximetry was not obtained. This case highlights the importance of co-oximetry in the emergency department for patients presenting with cardiopulmonary symptoms without obvious risk factors for acute cardiopulmonary disease. 

## Conclusions

This case was an unusual presentation of methemoglobinemia caused by topical dapsone, which persisted for 10 days in a patient without an identified underlying genetic disorder or other apparent risk factors. Although persistent methemoglobinemia due to oral dapsone has been described in the literature, it is not well described in patients only applying topical dapsone. While the 48-hour half-life of topical dapsone could explain the duration of symptoms, persistent methemoglobinemia has more often been reported in patients with elevated BMI or with poor renal function, and our patient had neither. The patient may have been experiencing significant oxidative stress from the methylene blue, but we cannot be certain as no lactate dehydrogenase was drawn during the initial admission. Her lactate dehydrogenase was elevated early in her second admission, but the level would also have been elevated in the setting of hemolysis. The patient's methemoglobinemia did resolve without further spikes in recorded levels once treated solely with ascorbic acid, which may support the theory that oxidative stress from the methylene blue was contributing to the duration of symptoms. Additionally, our patient went on to develop what was thought most likely a true autoimmune hemolytic anemia, given the rapid improvement in symptoms and lab values in the setting of high-dose prednisone despite a negative Coombs test. While hemolytic anemia has been seen after methemoglobinemia, it is usually in the setting of underlying G6PD deficiency. It is important to be aware that G6PD levels may be falsely elevated in the setting of hemolysis; therefore, repeat G6PD levels may be considered several months after the acute episode has resolved and the patient no longer requires medical treatment. Lastly, this case highlights the importance of co-oximetry testing in otherwise healthy young patients presenting with cardiopulmonary symptoms, including acute dyspnea, cyanosis, and decreased pulse oximetry unresponsive to supplemental oxygen. Furthermore, chocolate or dark-colored arterial blood should prompt clinicians to consider a methemoglobinemia diagnosis. It appears likely that our patient presented with a similar but less severe episode five months earlier, but no co-oximetry was obtained, which may have led to a missed diagnosis at that time.
